# CD169+ Subcapsular Macrophage Role in Antigen Adjuvant Activity

**DOI:** 10.3389/fimmu.2021.624197

**Published:** 2021-03-18

**Authors:** Christina Lisk, Rachel Yuen, Jeff Kuniholm, Danielle Antos, Michael L. Reiser, Lee M. Wetzler

**Affiliations:** ^1^ Section of Infectious Diseases, Department of Medicine, Boston Medical Center, Boston, MA, United States; ^2^ Department of Microbiology, Boston University School of Medicine, Boston, MA, United States; ^3^ Department of Microbiology and Immunology, University of Pittsburgh School of Medicine, Pittsburgh, PA, United States; ^4^ DRK-Blutspendedienst, BaWü-Hessen gGmbH, Frankfurt, Germany

**Keywords:** adjuvants, TLR-ligand based adjuvants, PorB, Neisseria, TLR2, antibody production, follicular dendritic cells, antigen deposition

## Abstract

Vaccines have played a pivotal role in improving public health, however, many infectious diseases lack an effective vaccine. Controlling the spread of infectious diseases requires continuing studies to develop new and improved vaccines. Our laboratory has been investigating the immune enhancing mechanisms of Toll-like receptor (TLR) ligand-based adjuvants, including the TLR2 ligand Neisseria meningitidis outer membrane protein, PorB. Adjuvant use of PorB increases costimulatory factors on antigen presenting cells (APC), increases antigen specific antibody production, and cytokine producing T cells. We have demonstrated that macrophage expression of MyD88 (required for TLR2 signaling) is an absolute requirement for the improved antibody response induced by PorB. Here-in, we specifically investigated the role of subcapsular CD169+ marginal zone macrophages in antibody production induced by the use of TLR-ligand based adjuvants (PorB and CpG) and non-TLR-ligand adjuvants (aluminum salts). CD169 knockout mice and mice treated with low dose clodronate treated animals (which only remove marginal zone macrophages), were used to investigate the role of these macrophages in adjuvant-dependent antibody production. In both sets of mice, total antigen specific immunoglobulins (IgGs) were diminished regardless of adjuvant used. However, the greatest reduction was seen with the use of TLR ligands as adjuvants. In addition, the effect of the absence of CD169+ macrophages on adjuvant induced antigen and antigen presenting cell trafficking to the lymph nodes was examined using immunofluorescence by determining the relative extent of antigen loading on dendritic cells (DCs) and antigen deposition on follicular dendritic cells (FDC). Interestingly, only vaccine preparations containing PorB had significant decreases in antigen deposition in lymphoid follicles and germinal centers in CD169 knockout mice or mice treated with low dose clodronate as compared to wildtype controls. Mice immunized with CpG containing preparations demonstrated decreased FDC networks in the mice treated with low dose clodronate. Conversely, alum containing preparations only demonstrated significant decreases in IgG in CD169 knockout mice. These studies stress that importance of subcapsular macrophages and their unique role in adjuvant-mediated antibody production, potentially due to an effect of these adjuvants on antigen trafficking to the lymph node and deposition on follicular dendritic cells.

## Introduction

Vaccines represent one of the greatest public health advancements of the last 50 years (1–5). However, there is still a great need for new vaccines for many infectious diseases including HIV, malaria, tuberculosis etc (6–10).. It is imperative that vaccine research continue in order to provide protection to these infectious diseases. One way in which vaccine research is progressing is the use of subunit vaccines (9, 11, 12). These vaccines consist of an antigen to protect against and an adjuvant to stimulate the immune response. Adjuvants can be divided up into five main groups – mineral salts, oil emulsion, microbial products, saponins, or synthetic products (13). The microbial product group contains a subclass of adjuvants that stimulate through pattern recognition receptors (PRR), more specifically Toll-like receptors (TLRs) (14–21). TLRs can be either extracellularly or intracellularly within the endosome (22–25). Depending on which TLR is engaged, cellular signal occurs leading to predictable downstream stimulation and effects. This predictableness makes TLR-ligand based adjuvants useful tools to investigate cellular pathways during an immune response (23, 26–28). Our lab has focused on these cellular mechanisms of adjuvants, especially PorB, the major outer membrane protein from *Neisseria meningitidis*, which signals through TLR2/1 heterodimers (29). PorB has been shown to significantly increase adaptive immune responses, such as antigen-specific antibodies (30, 31) and clearance of *Listeria monocytogenes via* CD8^+^ T cells (32). PorB has also been shown to stimulate antigen presenting cells and enhance adaptive immune responses by increasing their expression of co-stimulatory factors, increase their cytokine expression, and enhance their antigen cross-presentation (30, 33). Most recently, our lab has shown that PorB can also increase deposition of antigen on germinal center follicular dendritic cell (FDC) networks and can even increase the size of such networks (34).

It is well known that innate immune cells have the ability to influence and skew the adaptive immune responses in order to protect against pathogens (19, 35–38). The early induction events within the lymph node and spleen, which lead to germinal center formation and affinity maturation, remain topics of active research. Complete knowledge of such dynamics will lead to a better understanding of infection and prevention by utilizing the immune system. Recently our lab demonstrated that conditional knockouts of the TLR-signaling molecule MyD88 in macrophages specifically prevented the adjuvant effect of PorB as determined by a decrease in the production of OVA-specific IgG (31). Surprisingly, dendritic cells (DC) were not able to rescue the loss of MyD88 signaling within the macrophages. The work presented here which specifically examines the role of subcapsular sinus (SCS) and marginal zone macrophages in improved immune responses after vaccination using various adjuvants, is a follow-up to these studies, as the SCS or marginal zone macrophages have unique functions that DCs cannot perform (39, 40).

Multiple studies have indicated SCS macrophages in antigen and immune complex retention from the lymph, transportation into the lymph node (39–45), and activation of the adaptive immune response including antigen deposition onto follicular dendritic cells (46), antibody production (47) and CD8^+^ T cell activation (48–50). These studies have determined that SCS macrophages exploit their location within the lymph node, their unique cellular properties, and the expression of singlec 1 (CD169) receptor on the cell surface in order to influence early immune induction events (51–53). The studies described here provide critical information about the potential role of these macrophages in the effect of vaccine adjuvants, including both TLR-ligand based adjuvants (PorB and CpG) and particulate-based adjuvants (Alum) and/or the requirements of CD169 expression for the effects and enhancement of antigen specific antibody production.

## Methods

### Animals

Four to eight weeks old C57Bl/6J (referred to as ‘wild type’, stock #000664) mice were obtained from Jackson laboratories (Bar Harbor, ME). All mice were maintained within the Laboratory Animals Science Center (LASC) at Boston University School of Medicine under Dr. Lee Wetzler’s animal protocol 14155. The Boston University Institutional Animal Care and Use Committee (IACUC) approved all research conducted using animal models.

CD169 knockout mice were a gift from Dr. Paul Crocker, University of Dundee. Polymerase chain reaction (PCR) was performed on these animals to ensure the genotype was correct.

### Genotyping for CD169 Global Knockout

CD169 knockout animals were ear punched after weaning for genotyping. RedExtract-N-Amp Tissue PCR Kit (Sigma, Cat#E7526) was used according to manufacturer's protocol. PCR reaction was prepared using RedExtract-N-Amp Tissue PCR Kit (Sigma, Cat#R4775) according to manufacturer’s protocol.

To determine the effectiveness of the CD169 KO, the following PCR protocol was used. CD169 Primers: Forward - CAC CAC GGT CAC TGT GAC AA, Reverse - GGC CAT ATG TAG GGT CGT CT. Both primers are used at a final concentration of 1µM with the following PCR program:1. 92°C for 2:00, 2. 92°C for 0:30, 3. 57°C for 0:30, 4. 72°C for 1:30, 5. Repeat step #2 x35, 6. 72°C for 5:00, 7. Store at 4°C. When the transgene is present the expected product is 1,700 bp, as compared to 486 bp in WT mice.

### Specificity of Clodronate for Subcapsular Macrophages

Clodronate (Liposoma Research, SKU:C-005) treated animals received intravenous (IV) tail vein injections with different doses of clodronate – either high dose (40 mg/kg) or low dose (6.5 mg/kg). Low dose clodronate has been previously published to deplete the subcapsular subtypes of macrophages within the lymph node (54). Twenty-four hours post IV injection, flow cytometry and immunohistochemistry (IHC) was used to determined depletion of CD169^+^ macrophages. Briefly, inguinal lymph nodes were isolated and placed in cold PBS for flow cytometry analysis. Iliac lymph nodes were isolated and placed in molds (ThermoFisher) with optimal cutting temperature (OCT) medium (Richard Allan Scientific). These samples were frozen on dry ice for immunohistochemistry (IHC).

### Vaccine Regime

Wild type and CD169 knockout mice between the ages of four to eight weeks were immunized subcutaneously with 100 µl vaccine using a 28G insulin syringe (Becton Dickinson Cat# 3294161). Vaccine groups consisted of PBS (vehicle control), ovalbumin (OVA, 10 µg), OVA + PorB (10 µg), OVA+ CpG (10 µg, Invivogen, Cat# ODN1826), and OVA+ aluminum salts (alum, 200 µg Sigma, Cat# A8222). Clodronate treated groups received an IV tail vein injection one day prior to subcutaneous vaccinations. For immunoglobulin (IgG) studies, mice were vaccinated three times, two weeks apart as shown in **Supplemental Figure 3**. Two weeks after the final injection, the animals were sacrificed and the blood collected for antigen (ovalbumin, OVA) specific immunoglobulin ELISAs (n=6–12). The concentrations and volumes for vaccines were from previously determined publications (30). For antigen deposition onto follicular dendritic cells, mice were vaccinated subcutaneously and euthanized 24 h post injection (**Supplemental Figure 4**). Vaccination groups consisted of ovalbumin (OVA) fluorescently labeled with Alexa 594 (OVA-A594, Life technologies) alone, OVA-A594 + PorB, OVA-A594 + CpG, or OVA-A594 + Alum. Draining lymph nodes were isolated for IHC and flow cytometry analysis.

### PorB Isolation

Porin B (PorB) was isolated as previously published (55).

### Flow Cytometry for CD169^+^ Macrophages and Follicular Dendritic Cells

Draining lymph nodes were isolated for flow cytometry and placed in cold PBS immediately after isolation. Single cell suspensions were prepared as follows unless indicated otherwise. Tissues were pushed through a 70 µm cell strainer. Samples were then incubated for 3 min in ACK (150 mM NaH_4_CL, 50mM KHCO_3_) buffer to lysis red blood cells. The samples were then washed in PBS and re-filtered through a 70 µm cell strainer. At this point, samples were counted and stained for flow cytometry. Cells were incubated with a live/dead stain (Biolegend, Cat#423105) for 30 min, in the dark at 4°C. Cells were then washed with 5x FACSBuffer (PBS, 0.5%BSA, and 2% EDTA) and spun down at 1,600 rpm at 4°C. Cells were then incubated with CD16/CD32 Fc block (eBioscience, 48-0032-82) for 10 min in the dark at room temperature. Cells were then plated in a 96 V-well bottom plate (Corning, CLS3896-48EA), spun down at 1,600 rpm at 4°C and stained for flow cytometry. All dilutions were 1:200 unless noted. For subcapsular macrophages analysis, antibodies included: CD169-FITC (Bio-Rad, 0308), CD11b–PE (Biolegend, 123128), CD19-BUV395 (BD Horizon, 563557), F4/80–PerCP5.5 (Biolegend, 123128) CD3–eFlour (Invitrogen, 48-0032-82), CD11c–APC (BD Pharmigen, 550261). Gating strategy is shown in **Supplemental Figure 2A**. A fluorescence minus one (FMO) stain was used where all the antibodies in the panel are present with the exception of CD169 as shown in **Supplemental Figure 2B**. For follicular dendritic cells, single cell suspensions were prepared as previously reported (56) and is briefly described. Draining lymph nodes were placed in cold PBS and manually minced on a petri dish with a scalpel. The samples were transferred to a 24-well plate (FisherScientific, Cat #08-772-1H), incubated with DMEM containing 2% FBS (ThermoFisher, Cat#26140079), 33.3 mg/ml collagenase type IV (ThermoFisher, Cat#17104019), and 2,500 U/ml DNase I (ThermoFisher, Cat#18047019). Samples were incubated for 1 h at 37°C. After which, the samples were strained through 70 µm filter, although not pushed through to ensure the integrity of the FDCs remained intact. Cells were spun down at 1200 rmp at 4°C and then stained as described above for flow cytometry. All antibody dilutions were 1:200 unless otherwise noted. CD21/CD35 – BV421, CD45 – APC, CD19 – BUV395 (1:400), ICAM-1 – FITC. Gating strategy is shown in **Supplemental Figure 5A**. A fluorescence minus one (FMO) was stained for all colors within the panel excluding CD21/CD35 shown in **Supplemental Figure 5B**. All samples were analyzed on an LSRII, a machine available within the Boston University flow core, on a low flow setting to ensure the integrity of FDC remained intact.

### Immunohistochemistry for Subcapsular Macrophages and Antigen Deposition onto Follicular Dendritic Cells

For specificity of clodronate, lymph nodes were isolated 24 h after IV injections of either vehicle controls, low dose clodronate treatment (6.5 mg/kg), or high dose clodronate treatment (40 mg/kg). Draining lymph nodes were then put into molds containing optimal cutting temperature (OCT) medium (Richard Allan Scientific, Kalamazoo, MI, USA) and frozen on dry ice. Tissues were cut on a microm HM 550 (Microm International GmBH, Germany). 8 µm sections were obtained and placed on Colorfrost Plus slides and stored at -80°C until staining. For staining, sections were air dried for 15 min at room temperature, fixed in acetone at −20°C for 10 min, and air dried for 10 min at room temperature. Sections were re-hydrated in TBS buffer with 0.05% Tween-20 (TBST) then blocked for 30 min at room temperature with TBST with 5% BSA. Sections were rinsed with PBS and then stained with antibodies overnight at 4°C followed by three rinses with PBS. For clodronate specificity studies, the following antibodies and reagents were used: FITC hamster anti-mouse CD169 (Biolegend, Cat#142405) and F4/80 (Biolegend, Cat#123127). All antibodies were used at 1:200 dilution. For antigen deposition on FDCs, draining lymph nodes were isolated 24 h post subcutaneous injections and stained with conjugated (CD11c, Biolegend, Cat#117309) and primary (FDC-M1, BD Biosciences, Cat#551320) antibodies overnight at 4°C followed by three rinses with PBS. Secondary antibody (anti-rat 488, Biolegend, Cat#405) was added to the slides for 1 h at room temperature followed by three washed in PBS. Antibody concentration for the primary was 1:100. Conjugated and secondary antibodies were used at 1:200 dilution. Stained lymph node sections were mounted in Fluoroshield mounting medium with DAPI (Abcam) and dried overnight at room temperature. A Leica SP5 confocal microscope (Leica AG) was used to examine all sections using the Leica LAS AF the 10x and 63x oil immersion objectives. The images were arranged and analyzed using ImageJ (NIH).

### Enzyme-Linked Immunosorbent Assay (ELISA) for Antigen Specific Antibodies

Sera was collected at the time of euthanasia *via* heart sticks from all animals. Ninety-six well immulon 2 HB (ThermonFisher 3455) were coated with 5 µg/ml of OVA in carbonate buffer and incubated overnight at 4°C. Sera was diluted in tris-buffered saline and tween (TBST, 0.05% Tween) starting at 1:50. One hundred microliters of the dilutions were then added to the coated plates. A serial dilution for each sample was done horizontally across the plate. The plates were then incubated overnight in 4°C. The plates were then washed with 200 µl/well with 0.05% TBST three times. Alkaline phosphatase-conjugated anti-mouse total IgG or IgG1or IgG2c subclasses (Sigma Aldrich, St. Louis, MO) was added to each well and incubated at room temperature for 1 h. Total IgG was diluted 1:3,000 and subclasses for IgG was diluted to 1:2,000 in 0.05%TBST. Plates were then washed again and developed with one-step p-nitrophenyl phosphate (Pierce, Rockford, IL). The optical density was measure on BioTek Synergy HT and analyzed with the Gen5 software. For total IgG, colorimetric values were converted to nanograms/milligram from a standard curve created in ImageJ software as previously described (57). End point titers for IgG subclasses were determined by OD x dilution.

### Statistics

Statistics were calculated in GraphPad Prism (version 8.0). Differences in OVA-specific IgG and IgG subtypes in were calculated by T tests between wild type control animals and clodronate treated animal or CD169 knockout animals. ANOVA with Sidak’s multiple comparisons test was used for all other analysis. *p<0.05, **p<0.01, ***p<0.001, ****p<0.0001

## Results

### Description of Mouse Models Used for Antibody Production

In the studies describe below we used mice with defects in the subcapsular macrophages, either lacking the CD169 molecule or removal by treatment with low dose clodronate. CD169 global knockout animals were genotyped to ensure their genetic composition was correct. As shown in **Supplemental Figure 1**, the transgene was present at 1,700 base pair (bp) while the wildtype was at 468 bp. To complement our global knockout mouse, we utilized clodronate liposome treatment, which has been shown to deplete macrophages in the lymph nodes and the spleen by causing apoptosis from increased intracellular concentrations of clodronate. At higher doses (40 µg/kg), clodronate liposomes deplete both CD169^+^ macrophages and conventional (F4/80^+^) macrophages whereas lower dosages (6.5 µg/kg) result in the preferential depletion of CD169^+^ macrophages in mice (54). In order to demonstrate the specific depletion of these macrophages, fluorescently labeled antibodies recognizing CD169^+^ and F4/80^+^ expressing cells were used in flow cytometry and immunohistochemistry studies to examine the amount of CD169^+^ macrophages after high dose clodronate treatment, low dose clodronate treatment, and vehicle control animals. These results are shown in **Supplemental Figures 2C–G**. The specificity of low dose clodronate treatment to CD169^+^ macrophages allowed us to investigate the role of these cells in antibody production. The combination of these mouse models, CD169 knockout and low dose clodronate treatment, allowed for thorough investigations into the role of the subscapular (SCS) macrophages, as well as the expression CD169, in adjuvant mediated vaccine induced antibody production.

### CD169^+^ Macrophages and CD169 Expression are Necessary for TLR-Ligand Adjuvant-Dependent Antibody Production

**Figure 1:**
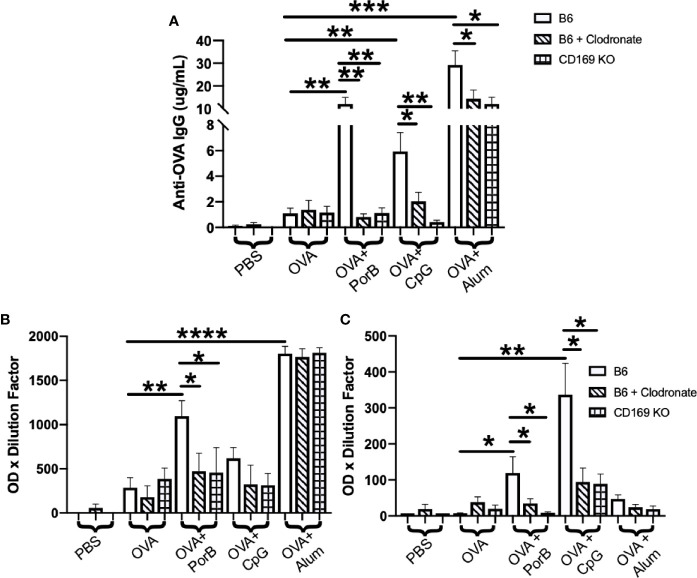
Antigen-specific immunoglobulin (IgG) is significantly decreased in immunized low dose clodronate treated mice and CD169 knockout mice **(A)** Total anti-ovalbumin (OVA)-IgG **(B)** anti-OVA-IgG1 and **(C)** anti-OVA-IgG2c levels were measure by enzyme-linked immunosorbent assay (ELISA) following immunizations with PBS, OVA alone, or OVA + PorB, OVA + CpG, or OVA + Alum. Wildtype (open bars), wildtype treated low dose clodronate (striped bars), or CD169 knockout mice (checkered bars) were immunized three times at 2-week intervals. The results shown are from sera collected 2 weeks after third immunizations. n = 7–14 per group. Statistics were calculated by ordinary one-way ANOVA with Sidak’s multiple comparisons test. *p < 0.05, **p < 0.01, ***p < 0.001 ****p < 0.0001.

We hypothesized that use of CD169 KO mice or depletion of CD169^+^ macrophages by low dose clodronate would adversely affect the ability of PorB, and possibly other adjuvants, to induce OVA specific antibodies. CD169 KO mice or low dose clodronate treated mice were immunized, per our protocol, and OVA-specific IgG was measured from the sera two weeks after the third immunization. These results are displayed in **Figure 1A**. Alum demonstrated the greatest increase in OVA-specific IgG, which was slightly diminished in low dose clodronate treated mice and CD169 knockout mice. Both TLR-ligand based adjuvants, PorB and CpG, also showed significant increases in total OVA-specific IgG in wild type animals, as expected (31), however IgG levels were greatly decreased in the in low dose clodronate treated mice and CD169 knockout mice.

To determine if the loss of CD169^+^ macrophages or the CD169 molecule influences IgG responses associated with Th1 or Th2 type responses, OVA-specific IgG subtypes in sera from the above-mentioned mice were analyzed by ELISAs. These data would provide insight on whether subcapsular macrophages and/or CD169 influence Th1 or Th2 responses. Th2 responses are characterized by IgG1 production while Th1 response are characterized by IgG2b and IgG2c production (58–60). PorB+ OVA and Alum + OVA immunized mice had significant increases in the OVA-specific IgG1 (**Figure 1B**). However, only low dose clodronate treated mice and CD169 KO mice that were immunized with PorB+OVA had significant decreases in IgG1 as compared to the WT mice (**Figure 1B**). WT mice immunized with either TLR-ligand adjuvant (PorB or CpG) had a significant increase in IgG2c production which was significantly decreased in mice treated with low dose clodronate and CD169 knockout animals.

### Adjuvant Induced Increases in Antigen Levels within Lymph Nodes is Influenced by Depletion of CD169^+^ Macrophages

**Figure 2:**
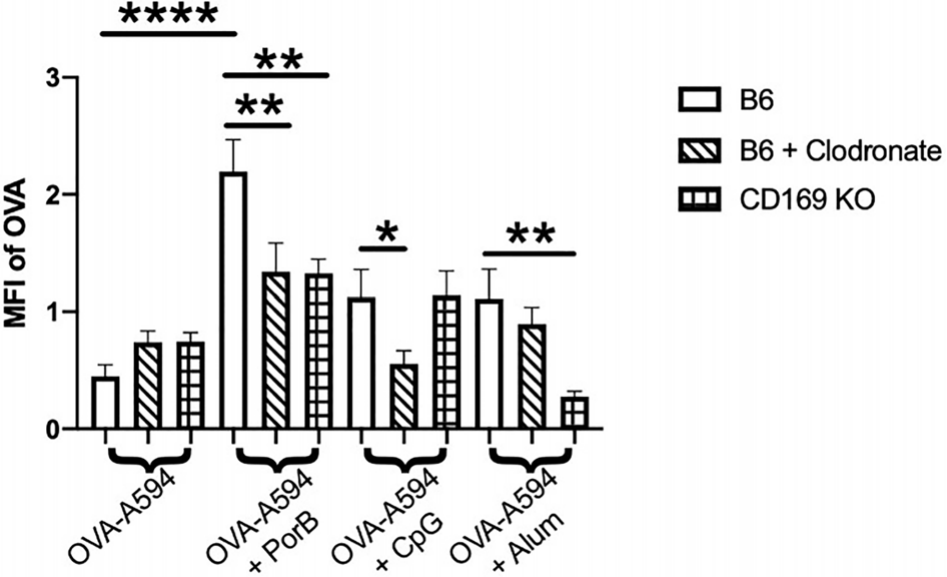
Adjuvant induced antigen presence in draining lymph nodes is affected in immunized low dose clodronate treated mice and CD169 knockout mice. Mean fluorescent intensity (MFI) of antigen (OVA) in draining lymph nodes 24 h post vaccination of either OVA-A594, OVA-A594+PorB, OVA-A594+CpG, or OVA-A594+Alum was measured *via* ImageJ. Wild type control injections are shown in the bars with no pattern. Low dose clodronate treated animals are shown in the striped bars. CD169 knockout mice are shown in checkered bars. MFI of OVA was quantified by the ImageJ measurement tool after the subtraction of the background. n = 8–13. Statistics were calculated by ordinary one-way ANOVA with Sidak’s multiple comparisons test. *p < 0.05, **p < 0.01, ****p < 0.0001.

The diminished antibody responses in total IgG and IgG subtypes led us to hypothesize that low dose clodronate treatment and CD169 knockout were lessening the immune stimulating ability of adjuvants. Antigen deposition on follicular dendritic cells within germinal centers is crucial for effective antibody production by improving B cell receptor affinity maturation (61, 62). To determine if adjuvants affects this process, the total amount of OVA present in the draining lymph nodes was measured 24 h post subcutaneous immunization with OVA with or without adjuvant preparations. As shown in **Figure 2**, PorB was the only adjuvant to significantly increase OVA mean fluorescent intensity (MFI) in the lymph nodes as compared to OVA alone, which was significantly decreased in low dose clodronate treated mice and CD169 knockout mice. A decrease was also seen with the use of CpG as an adjuvant, but only in mice treated with low dose clodronate. A similar decrease was seen with the use of alum but only in the CD169 KO mice.

### Follicular Dendritic Cell Networks Are Affected by Clodronate Treatment

**Figure 3:**
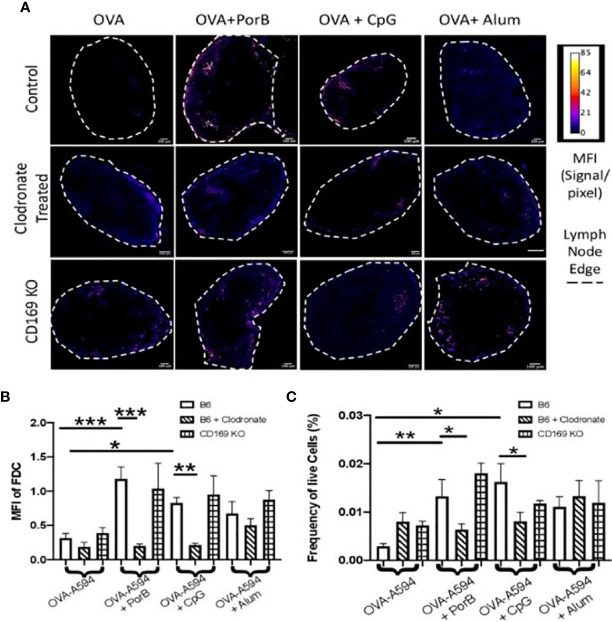
Toll-like receptor (TLR) ligand-adjuvant induced increases in the follicular dendritic cell (FDC) network are affected in low dose clodronate treated mice but not CD169 knockout mice. **(A)** Representative images for FDC networks in draining lymph nodes 24 h post adjuvant+OVA-A594 subcutaneous injections in mice treated with low dose clodronate 24 h prior to these immunizations or CD169 knockout mice. FDC expression is shown as a heat map where white designates the highest signal to pixel ratio and blue designates the lowest signal to pixel ratio. Scale bar is 100 µM. One out of two representative experiments are shown. **(B)** Mean fluorescence intensity (MFI) quantification from ImageJ of FDC in draining lymph nodes 24 h post injection of either OVA-A594, OVA-A594+PorB, OVA-A594+CpG, or OVA-A594+Alum in all three animal models. Wild type control injections are shown in the bars with no pattern. Low dose clodronate treated animals are shown in the stripped bars. CD169 knockout animals are shown in checkered bars. Multiple FDC networks were measured within the lymph nodes. n = 7–15/group. **(C)** Frequency of FDC cells in draining lymph nodes 24 h post subcutaneous injections with OVA-A594 +/- adjuvants. (n = 6). Gating strategy is shown in **Supplemental Figure 5**. Statistics were calculated by ordinary one-way ANOVA with Sidak’s multiple comparisons test. *p < 0.05, **p < 0.01, ***p < 0.001.

Although a decrease in antigen within the lymph nodes could explain a significant decrease in antibody production, it was also important to determine if depleting CD169 macrophages could also influence the follicular dendritic cell (FDC) network and antigen deposition on FDCs. These experiments follow up our previous published work demonstrating that PorB can greatly enhance both the FDC network and increase antigen deposition on FDCs (34). FDCs and FDC networks are essential for B cell receptor maturation and therefore antibody production. In addition, it has been shown that SCS macrophages are important for entry of particulate matter into the follicle and germinal center to interact with antigen presenting cells (APCs) and FDCs. **Figure 3A** displays representative images of draining lymph nodes for all treatments and immunization groups. FDC staining is shown as a heat map where the highest signal/pixel (px) ratio is shown as white and the lowest signal/px ratio is shown in blue. Similar to our previous work, only the use of TLR-ligand based adjuvants induced increased FDCs in the draining lymph node in immunized WT mice (top row). Interestingly, low dose clodronate treatment and removal of subcapsular macrophages diminished the adjuvant-induced increase of FDCs close to the baseline seen in unimmunized mice. In contrast, the use of CD169 KO mice did not affect the PorB induced increase in FDCs. Next, the MFI of FDC was quantified by the measurement tool within ImageJ (**Figure 3B**) and is consistent with IHC findings (**Figure 3A**). Finally, to further confirm these results, flow cytometric analysis of the draining lymph node were performed to compare the frequency and percentages of FDCs in similar treated and immunized mice. The data was consistent with the IHC and MFI measurements; immunizations including TLR-adjuvants significantly increased FDC frequencies in WT mice but was diminished in low dose clodronate treated mice and not in CD169 KO mice (**Figure 3C**).

### PorB Induced Increase of Follicular Dendritic Cell Antigen Deposition Is Diminished in Low Dose Clodronate Treated Mice and CD169 Knockout Mice

**Figure 4:**
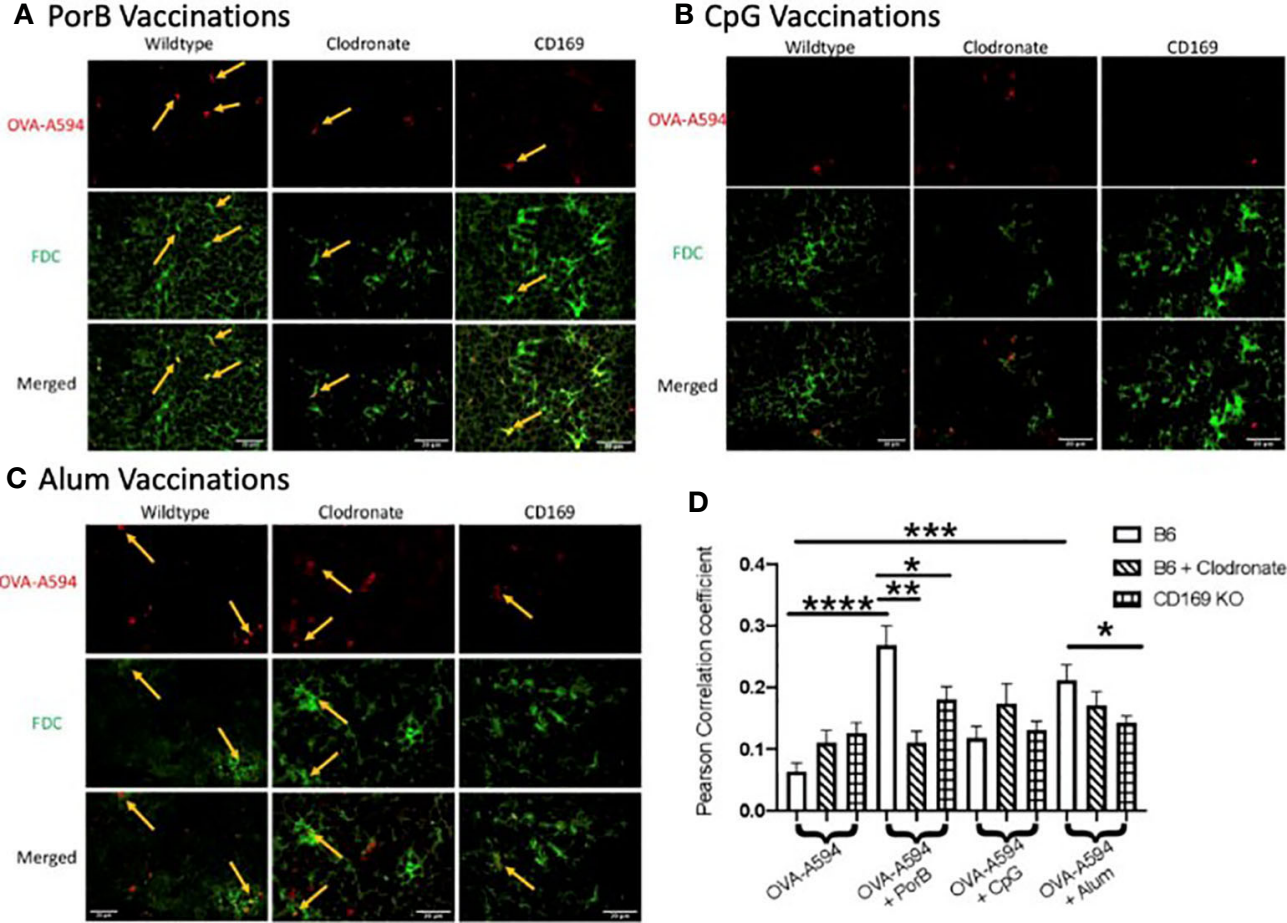
Adjuvant induced increase of antigen deposition on follicular dendritic cells (FDCs) is diminished in low dose clodronate treated mice and CD169 knockout mice. Representative immunohistochemistry images from draining lymph nodes from wildtype, clodronate treated mice, and CD169 knockout mice 24 h post subcutaneous immunizations of ovalbumin (OVA) with PorB **(A)**, CpG **(B)**, or Alum **(C)** and 48 h post clodronate treatment. FDC is shown in green. OVA-A594 is shown in red. Images were taken at 63x objective using a Leica SP5 microscope. Typical areas of co-localization are emphasized with yellow arrows. Scale bar represents 20 µM. One out of six representative experiments is shown. **(D)** Quantification of colocalization between fluorescently labeled OVA-A594 with FDC within draining lymph nodes 24 h post subcutaneous injections. Colocalization was assessed using Pearson Correlation coefficients calculated within JaCoP plugin in ImageJ after background subtraction and unsharp mask filter. n = 8–12/group. Wild type control injections are shown in the bars with no pattern. Low dose clodronate treated animals are shown in the stripped bars. CD169 knockout animals are shown in checkered bars. Statistics were calculated by ordinary one-way ANOVA with Sidak’s multiple comparisons test. *p < 0.05, **p < 0.01, ***p < 0.001 ****p < 0.0001.

Above, we demonstrated that PorB effects on FDC frequency was decreased in clodronate treated animals. These studies now determine whether the lack of CD169 macrophages affects PorB’s previously demonstrated increase of FDC antigen deposition (34) using Alexa-594 tagged (OVA-594). Consistent with our previous work, PorB induces an increase of colocalization of antigen with FDCs as shown by yellow arrows in **Figure 4A**. Interestingly, this colocalization was diminished when mice treated with low dose clodronate or CD169 KO mice were used (**Figure 4A**). Immunizations using Alum as an adjuvant significantly increased FDC/OVA colocalization in WT mice which was decreased in CD169 KO (**Figure 4C**). Vaccines that contained CpG showed no significant differences in colocalization between FDC and OVA in any of the animal models tested (**Figure 4B**).

### OVA Association With DCs and FDCs is Influenced by Depletion of SCS Macrophages

**Figure 5:**
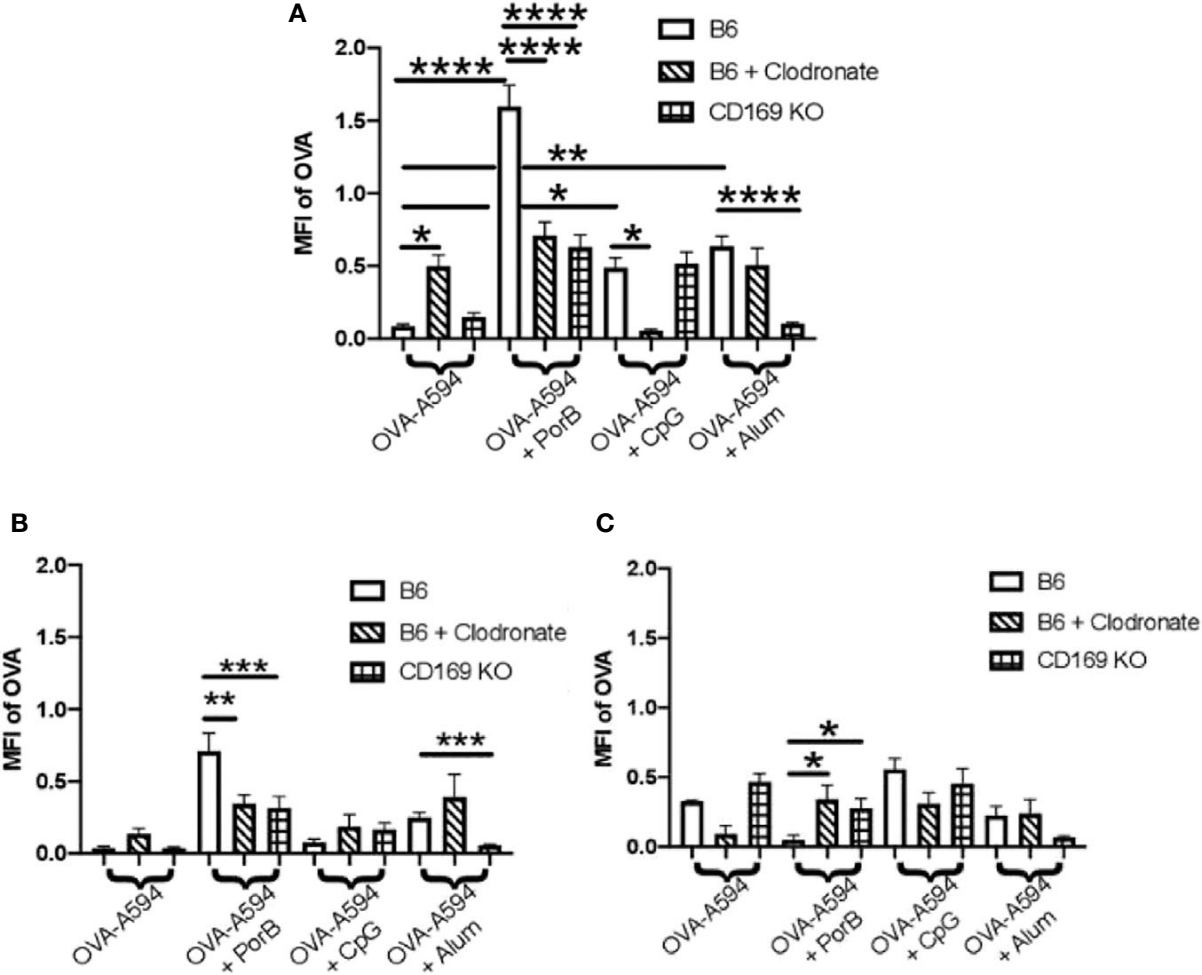
PorB-induced antigen association with dendritic cells (DCs) and follicular dendritic cells (FDCs) is diminished in low dose clodronate treated mice and CD169 knockout mice. These graphs display ovalbumin (OVA)-A594 association with DC and FDC and unassociated OVA within draining lymph node 24 h post subcutaneous immunizations of indicated adjuvants with OVA in WT mice, low dose clodronate mice of CD169 knockout mice. Injection of OVA without adjuvants was used as a control. MFI of OVA=594 associated with DC **(A)**, FDC **(B)**, or unassociated OVA **(C)** was assessed using the JaCoP plugin within ImageJ and determining the Mander’s correlation coefficient after subtracting the background and using an unsharp mask filter. The correlation coefficient is the percentage of OVA associated with either FDCs or DCs. This percentage was then multiplied by the total MFI of OVA within the lymph node (**Figure 2**) to determine MFI of OVA associated with DCs or FDCs. Wild type control injections are shown in the bars with no pattern. Low dose clodronate treated animals are shown in the striped bars. CD169 knockout animals are shown in checkered bars. Statistics were calculated by ordinary one-way ANOVA with Sidak’s multiple comparisons test. n = 5–7 *p < 0.05, **p < 0.01, ***p < 0.001, ****p < 0.0001.

To determine the distribution of antigen, Mander’s correlation coefficients were determined *via* JaCoP. This correlation coefficient allows for spilt channels of correlation to be determined (63) as percentages. We measured the percentages of OVA correlated to either dendritic cells (DCs) or FDC. This percent was then multiplied by the MFI of OVA within the lymph node from **Figure 2** to determine the percent of OVA associated with DCs, FDC, or unassociated. We defined “unassociated OVA” as the remaining percentage of OVA that was not associated with either DC or FDC (Unassociated OVA = 1-[MFI of OVA x (Mander’s coefficient for OVA/DC + Mander’s coefficient for OVA/FDC)]). There are, however, other cell types within the lymph node during this time with which OVA could associate (B cells or macrophages), though unlikely. For the purpose of these studies, these cell types are included in the “unassociated” section because of the importance of antigen loaded DCs and antigen deposited on the FDCs at early timepoints for induction of an adaptive immune response. All adjuvanted immunizations induced increase in OVA associated DCs, and consistent with our previous data (33), PorB induced the greatest association (**Figure 5A**). In both low dose clodronate treated mice and CD169 KO mice immunized with OVA + PorB, there were significant decreases in DC/OVA (**Figure 5A**) and FDC/OVA correlation coefficients (**Figure 5B**). Unassociated OVA in both low dose clodronate and CD169 knockout animals had a significant increase over WT mice in animals that received PorB + OVA vaccines (**Figure 5C**). CpG + OVA immunized mice only showed significant differences in DC/OVA correlation in clodronate treated animals (**Figure 5A**). No differences were seen with CpG + OVA immunizations in low dose clodronate treated mice or CD169 KO mice in FDC/OVA (**Figure 5B**). Alum adjuvant usage showed a significant decrease in both DC/OVA (**Figure 5A**) and FDC/OVA in CD169 KO mice (**Figure 5B**) but had no difference in low dose clodronate treated mice. Overall, the increases of OVA association with FDCs or DCs was always greater in mice receiving PorB + OVA immunizations, and the decrease in the low dose clodronate treated mice or CD169KO mice were subsequently greater when PorB was used as an adjuvant as opposed to CpG or Alum.

## Discussion

These studies utilized two animal models which interrogate the subcapsular macrophage population in lymph nodes and germinal centers to further characterize cellular mechanisms of adjuvant activity within these structures. Marginal zone and subcapsular macrophages were targeted for analysis for a number of reasons. Our lab has previously demonstrated that a defect in TLR signaling in macrophages, by cell specific genetic depletion of MyD88, greatly diminished the adjuvant effect of PorB (31). This data also demonstrated that no other antigen presenting cell could rescue the depleted antibody production seen in these mice. This focused our attention on macrophage subtypes whose function could not be replaced by dendritic cells or B cells, namely subcapsular and marginal zone macrophages. Importantly, Cyster et al. have shown that subcapsular macrophages are essential for antigen transport and deposition on FDC (64). Our recent publication demonstrates that PorB enhances these processes (34), further implicating the probable involvement of these cells in PorB’s adjuvant activity.

To investigate the role of these macrophages in adjuvant activity, two mouse models with altered subcapsular macrophages were utilized: 1) mice treated with low dose clodronate which has been shown to cause apoptosis and removal solely of SCS macrophages (65) and 2) CD169 global knockout (KO) mice. It is important to note that while clodronate treatment cause a depletion of a subcapsular macrophages, these cells are still be present in the CD169 KO mice, but do not express CD169 molecule. Immunization with adjuvanted vaccines in both types of mice demonstrated a decreased total antigen-specific antibody response as compared to wild type mice and this decreased was much more dramatic when TLR-ligand based adjuvants, PorB and CpG were used as compared to the use of Alum (**Figure 1A**).

The effects on antigen specific IgG subtypes induced by these adjuvants were investigated to determine if loss of SCS macrophages or CD169 could influence on Th1 or Th2 adaptive immune responses. Alum adjuvanted vaccines almost exclusively induced IgG1 [as expected (66)] which was unaffected in low dose clodronate treated mice and CD169 KO mice (**Figure 1B**), suggesting IgG1 antibody production associated with Alum is not dependent on SCS macrophages or CD169 expression. In contrast, when PorB was used as an adjuvant, OVA-specific IgG1 showed a significant decrease in both clodronate treated and CD169 KO mice as compared to WT mice (**Figure 1B**). The use of CpG as an adjuvant did not induce detectable levels of OVA-specific IgG1. Both TLR-ligand based adjuvants, PorB and CpG, induced OVA-specific IgG2c levels which were significantly decreased in clodronate treated and CD169 KO mice as compared to WT mice (**Figure 1C**). These data emphasize that TLR-ligand based adjuvants likely utilize SCS macrophages to a much greater extent than alum for their adjuvant effect. This is consistent with our MyD88 conditional KO data, as TLR2 and TLR9 both require MyD88 for signaling. To confirm the role of MyD88 in the adjuvant effect of PorB and CpG on these SCS and marginal zone macrophages, future studies will use MyD88 conditional KOs in these CD169^+^ cells utilizing MyD88-floxed mice and CD169-cre-recombinase mice.

As part of these studies, we investigated the potential association of SCS macrophages in relation to B cell receptor affinity maturation and somatic hypermutation which occurs in the germinal center and requires the use of FDCs. The quality of FDCs and the ability of adjuvants to enhance intact antigen deposition on FDCs were investigated. We have recently shown that PorB can increase intact OVA deposition on FDCs (34). As a follow-up, the effect of lacking SCS macrophages or CD169 expression on this process was examined utilizing low dose clodronate treated mice and CD169 KO mice. Results demonstrate that the adjuvants tested here (PorB, CpG, and alum) utilize SCS macrophages by different mechanisms. PorB’s effect on trafficking of OVA was greatly affected by loss of either marginal zone macrophages or the global loss of CD169 (**Figure 2**). Additionally, antigen deposition on FDCs (**Figure 4A**) was significantly decreased in both sets of mice as compared to WT groups. In combination, these findings define a mechanism to explain the diminution of OVA specific IgG production in these mice when immunized with PorB + OVA (**Figure 1**). These data also suggest that the adjuvant activity of PorB is likely more dependent on SCS macrophages for enhancing antibody production, when compared to CpG or alum. One explanation is that the native trimeric structure of PorB, which is preserved for these immunizations studies (55, 67), is similar to other particles that have been shown to influence SCS macrophages. Saunderson et al. have shown that exosomes are retained in the SCS macrophages, but in CD169 KO mice, these exosomes now progress into the paracortex of the lymph node (68). We hypothesize that PorB stimulates the marginal zone macrophages (in a MyD88-mediated manner) to a much greater extent than CpG or alum, leading to increased amounts of antigen within the lymph node and increased FDC antigen deposition. Studies examining the direct effect of PorB on SCS macrophages, including the potential role of TLR2 expression and MyD88 are planned to investigate this hypothesis.

Immunizations containing CpG showed significant increases in total OVA-specific IgGs, especially OVA-specific IgG2c (**Figure 1**) which was decreased in low-dose clodronate treated and CD169 KO mice, similar to PorB. However, though also a TLR-ligand based adjuvant, the effect of CpG on antigen levels in the germinal centers, the alterations in the FDC network and antigen deposition on FDCs and results in mice lacking SCS macrophages or CD169 expression was shown to be different as compared to PorB. These differences included a lack of increase in antigen deposition on FDCs (**Figure 3**), a lack of an effect on OVA germinal center association in CD169 KO mice (though decreased in the clodronate treated mice, **Figure 4B**). Similarly, there was a lack of an effect on OVA association with DCs in CD169 KO mice, but significantly decreased in the clodronate treated mice (**Figure 5**). This suggests a different role of these macrophages in the adjuvant activity for CpG which can be discerned in future studies.

To determine if non-TLR ligands have distinctive cellular mechanisms of adjuvanticity as compared to TLR-ligand based adjuvants, we included alum in our studies. Similar to PorB, our results revealed that alum + OVA injections induced total antigen specific IgG was significantly decreased in both low dose clodronate treated and CD169 KO mice when compared to wildtype controls (**Figure 1A**); however, these decreases, were not as robust or significant as the decreases seen when TLR-ligand based adjuvants, PorB and CpG were used. Interestingly, IgG1 subtype levels were similar in the SCS modified mice as compared to WT mice when alum was used as an adjuvant (**Figure 1B**). While investigating innate immune responses to determine if SCS macrophages or CD169 knockout animals influenced alum adjuvanticity, we have found that less antigen is present in the draining lymph nodes of CD169 knockout animals when compared to wildtype (**Figure 2**), including a decrease in antigen deposition onto FDC (**Figures 4C, D** and **5B**) as well as a decrease in OVA associated with DC (**Figure 5A**). Together these data suggests that alum requires expression of CD169 molecule for its adjuvanticity and its ability to allow antigen to enter secondary lymphoid organs, but PorB’s adjuvant activity appears to be more reliant on these cell types as lack of SCS macrophages causes a much greater decrease.

In conclusion, in examining the possible role of subcapsular sinus macrophages and/or the expression of CD169 on these cells we have found that these alterations affects the adjuvant effect of PorB, CpG, and alum but the effects were dependent on the adjuvant used. Interestingly, TLR-ligand based adjuvants had a more dramatic decrease than that of alum immunizations on antibody production. Investigating antigen within the draining lymph node, as well FDC and DC cellular association of antigen, we concluded that these three adjuvants work through different cellular interactions. PorB had the most dramatic defects in both clodronate treated and CD169 knockout mice, leading us to hypothesize that the extracellular TLR1/2 agonist utilizes the SCS macrophages more than the other adjuvants tested here. Since SCS macrophages have been shown to be pivotal for antibody production (69), we believe these data support that PorB has unique characteristics, such as the ability to stimulate both Th1 and Th2 antibody responses and the trimeric structure of the adjuvant, that make PorB a higher quality adjuvant than others.

## Data Availability Statement

The raw data supporting the conclusions of this article will be made available by the authors, without undue reservation.

## Ethics Statement

The animal study was reviewed and approved by Boston University IACUC.

## Author Contributions

The work was performed by CL, with help and advice from RY, JK, and MR and technical help from DA. All the work was planned with LW and CL. CL and LW wrote and edited the manuscript. All authors contributed to the article and approved the submitted version.

## Funding

These studies were funded by NIH Grant AI0404944 (LW). CL was funded by the Boston University Training Program in Inflammatory Disorders T32AI138933.

## Conflict of Interest

The authors declare that the research was conducted in the absence of any commercial or financial relationships that could be construed as a potential conflict of interest.
