# Supplementary material for: CD169+ Subcapsular Macrophage Role in Antigen Adjuvant Activity
Source: Front Immunol. 2021 Mar 18;12:624197. doi: 10.3389/fimmu.2021.624197 (PMC8012505; doi:10.3389/fimmu.2021.624197)
Supplement: Supplementary file 1 [file DataSheet_1.pdf]

Supplemental Figure 1: CD169 Knockout PCR gel

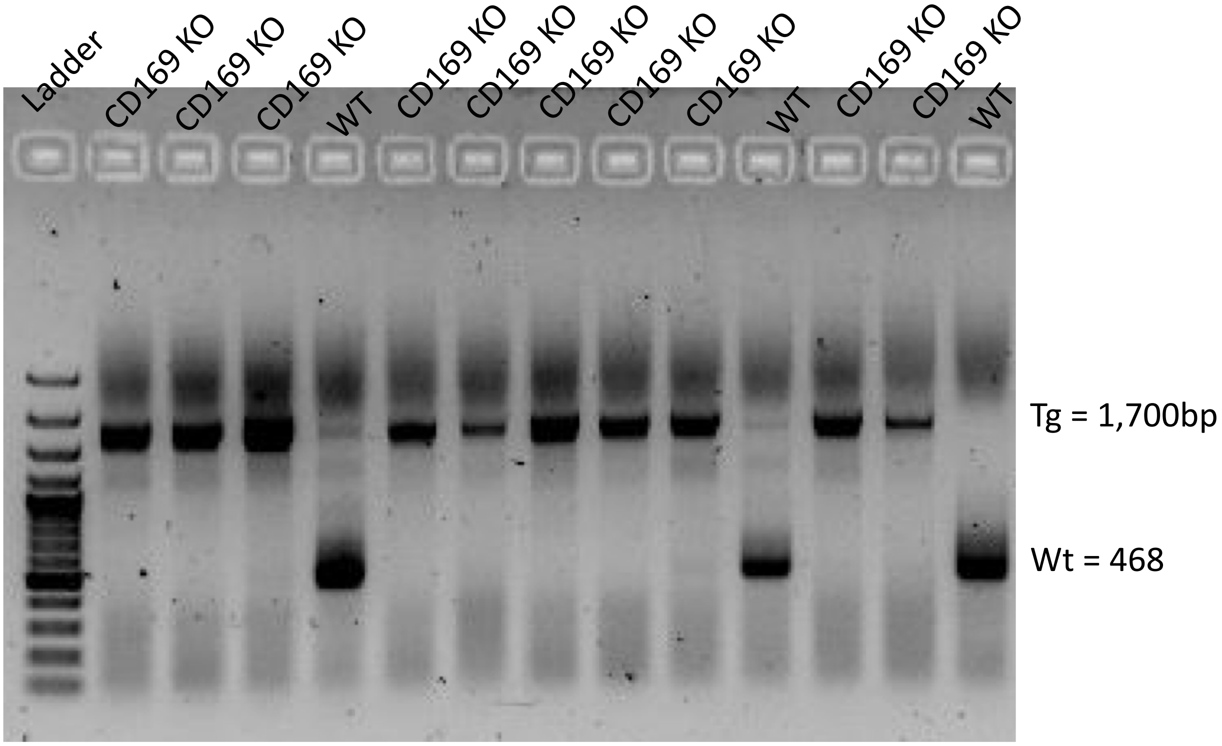

Supplemental Figure 1: CD169 PCR gel. Transgene (Tg) for CD169 is shown at 1,700 bp. Wildtype (Wt) is shown at 468 bp.

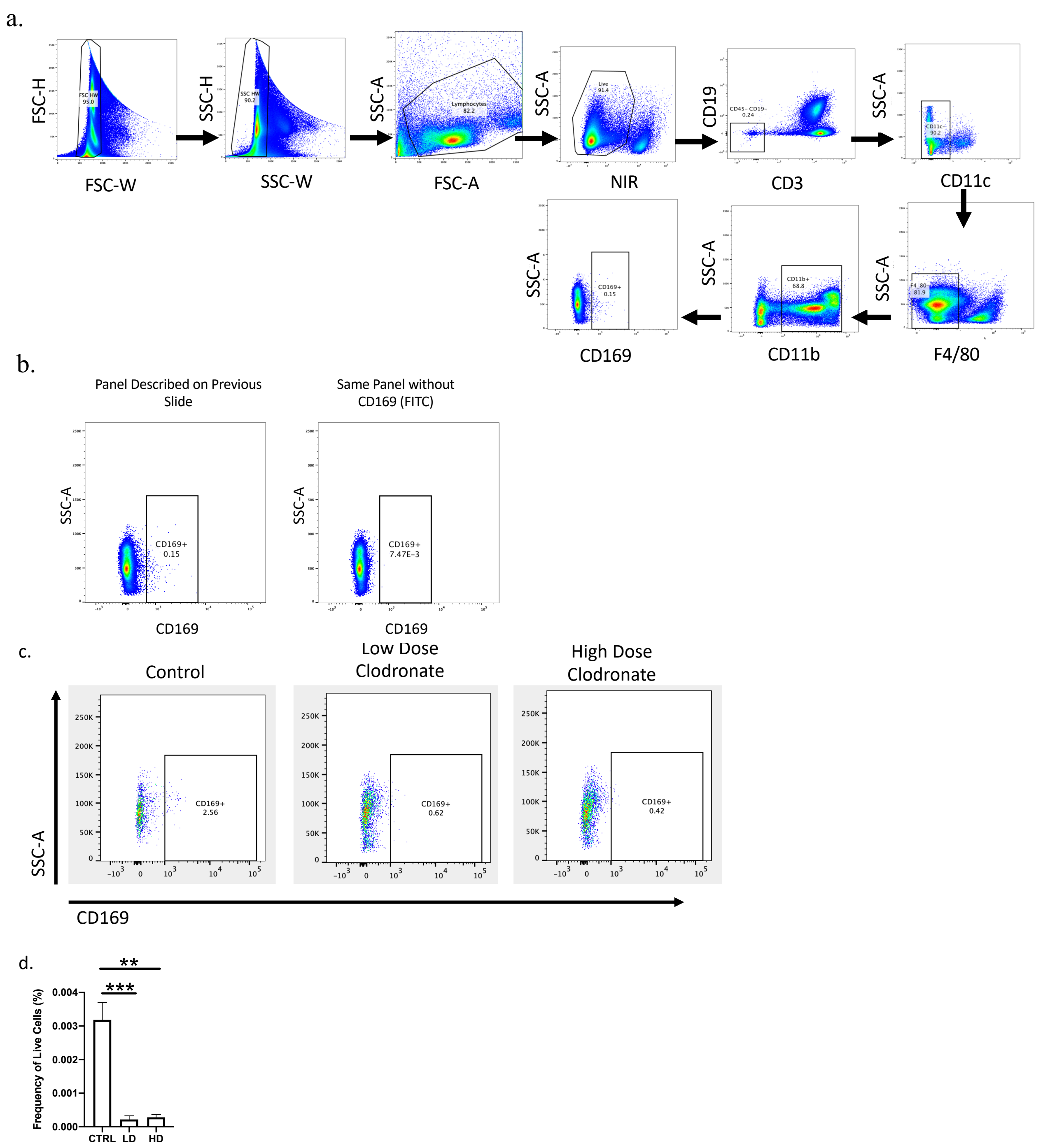
a.
FSC-H
FSC-W
FSC HW
95.0
SSC-H
SSC-W
SSC HW
90.2
SSC-A
FSC-A
Lymphocytes
82.2
SSC-A
NIR
Live
91.4
CD19
CD3
CD45- CD19-
0.24
SSC-A
CD11c
CD11c-
90.2
SSC-A
F4/80
F4_80
81.9
SSC-A
CD11b
CD11b+
68.8
SSC-A
CD169
CD169+
0.15
b.
Panel Described on Previous Slide
Same Panel without CD169 (FITC)
SSC-A
CD169
CD169+
0.15
CD169+
7.47E-3
c.
Control
Low Dose Clodronate
High Dose Clodronate
CD169+
2.56
CD169+
0.62
CD169+
0.42
SSC-A
CD169
d.
Frequency of Live Cells (%)
CTRL
LD
HD
**
***

e.

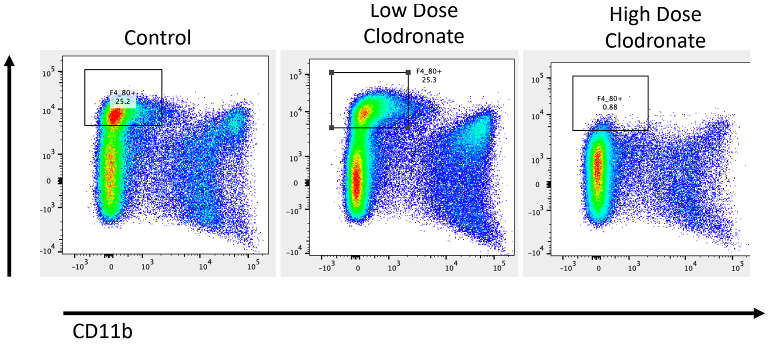

f.

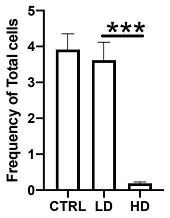

g.

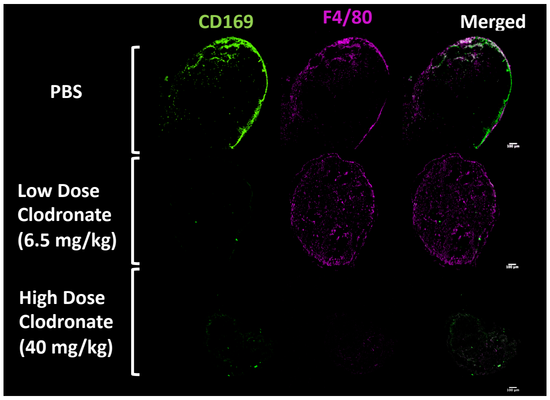

Supplemental Figure 2a. Gating strategy for CD169$^+$ macrophages in lymph nodes. Supplemental Figure 2b: Fluorescence minus one (FMO) for CD169. Supplemental Figure 2c and d: (c) Final gate from strategy shown in Figure 4.2a of vehicle control, low dose clodronate treated (6.5mg/kg) , or high dose clodronate (40mg/kg) treated animals. Y-axis shows side scatter. X-axis shows CD169. (d) Quantification from inguinal lymph nodes of animals treated with vehicle control, low dose clodronate treated (6.5mg/kg) , or high dose clodronate (40mg/kg). n = 5. **p<0.01 ***p<0.001Supplemental Figure 2e and f: (e) Gating for F4/80$^+$CD11b$^-$ events in inguinal lymph nodes 24 hours post vehicle control, low dose clodronate treated (6.5mg/kg) , or high dose clodronate (40mg/kg) treated animals. Y-axis shows F4/80. X-axis shows CD11b. (f) Quantification from inguinal lymph nodes of animals treated with vehicle control, low dose clodronate treated (6.5mg/kg) , or high dose clodronate (40mg/kg). n = 5. ***p<0.001Supplemental Figure 2g: Effect of clodronate on macrophages populations. Iliac lymph nodes were stain for CD169 (green) and F4/80 (purple) 24 hours post intravenous injection with either PBS, low dose clodronate (6.5 mg/kg), or high dose clodronate (40 mg/kg). Images were taken at 10x objective on a Leica SP5 microscope. Images shown are representative. Scale bar = 100um

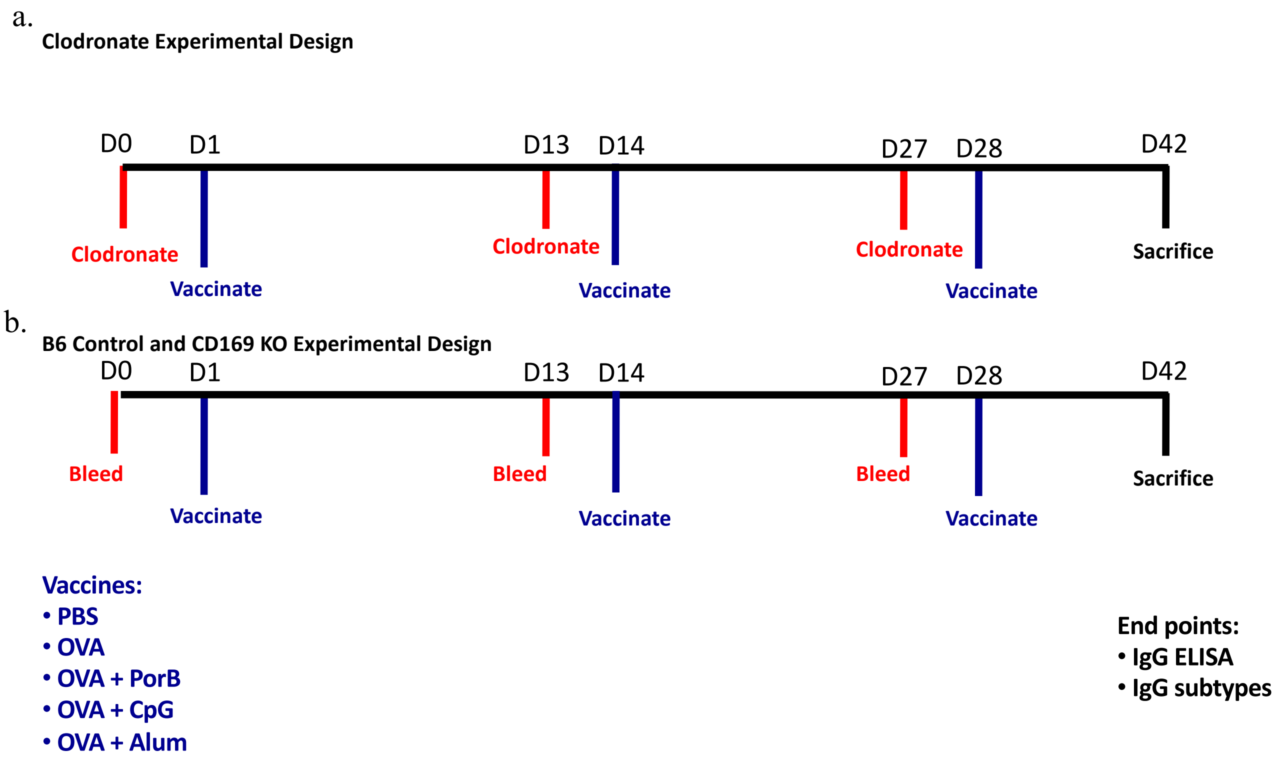

Supplemental Figure 3. Low dose clodronate treatments included in vaccine regime decreases antibody production. (a) Low dose clodronate and immunization schedule. This schedule was modified from a previously published immunization schedule from our lab. Mice were divided into 2 groups – one group receiving low dose clodronate treatment one day prior to immunization and the other group receiving encapasomes control. These groups were further divided into PBS, OVA, OVA+ PorB, OVA+ CpG, or OVA+Alum. Mice received subcutaneous immunizations 3 times, with 2 weeks in between immunizations.

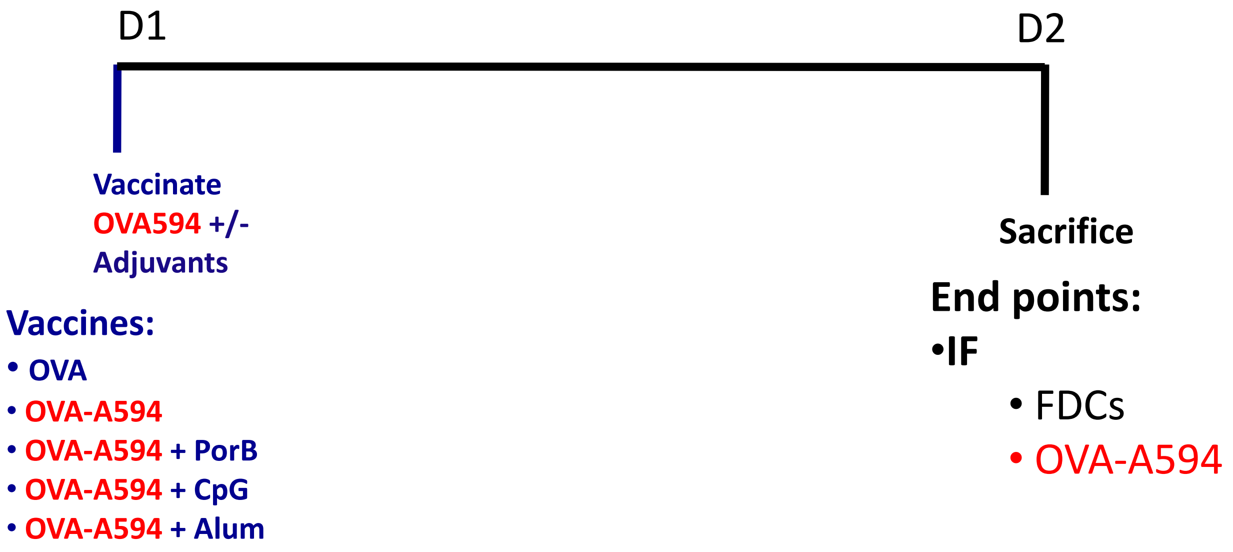

Supplemental Figure 4. Experimental design for antigen trafficking within the lymph nodes. mice were divided into OVA, OVA-A594, OVA-A594 + PorB, OVA-A594+ CpG, or OVA-A594+Alum. Mice received subcutaneous injection.

a.

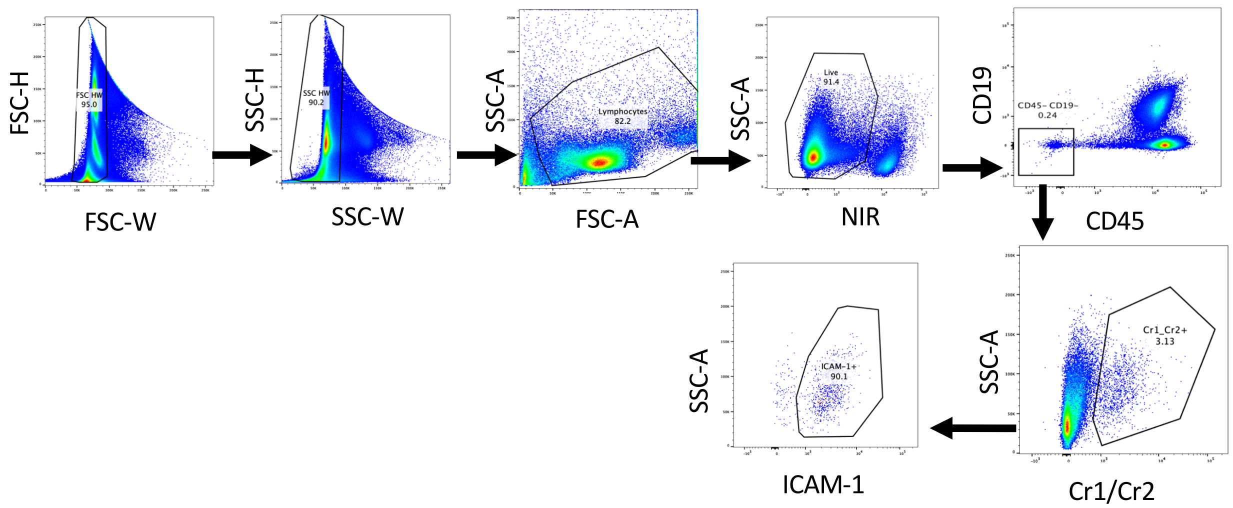

b.

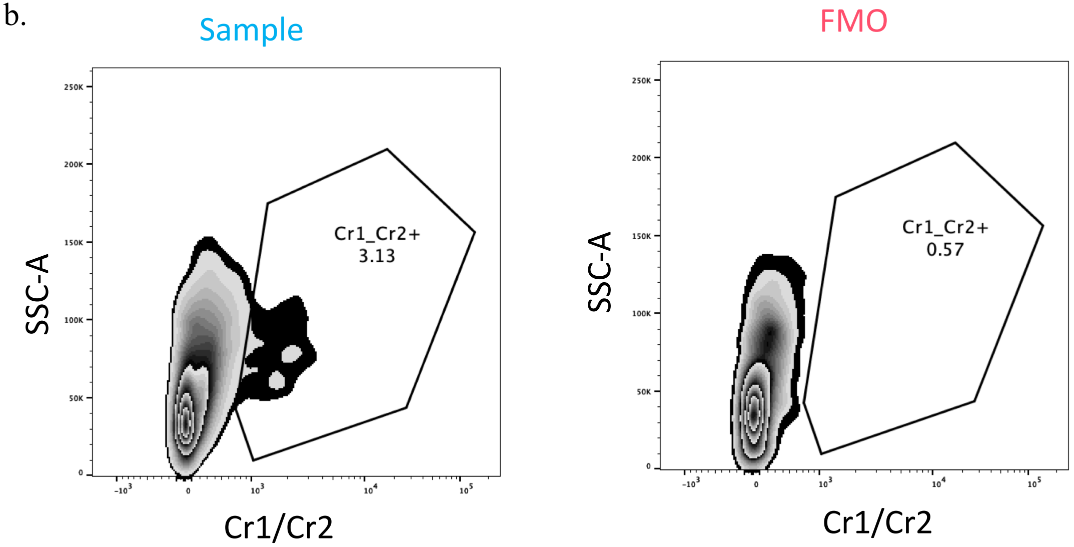

Supplemental Figure 5: (a) Gating strategy for follicular dendritic cells (FDCs). (b) Fluorescence minus one (FMO) for Cr1/Cr2. Two samples were analyzed based on the gating strategy in Supplemental figure 5 a. One sample contained Cr1/Cr2 antibody (pictured on the left). The other did not (pictured on the right). These are the results of the flow cytometry.

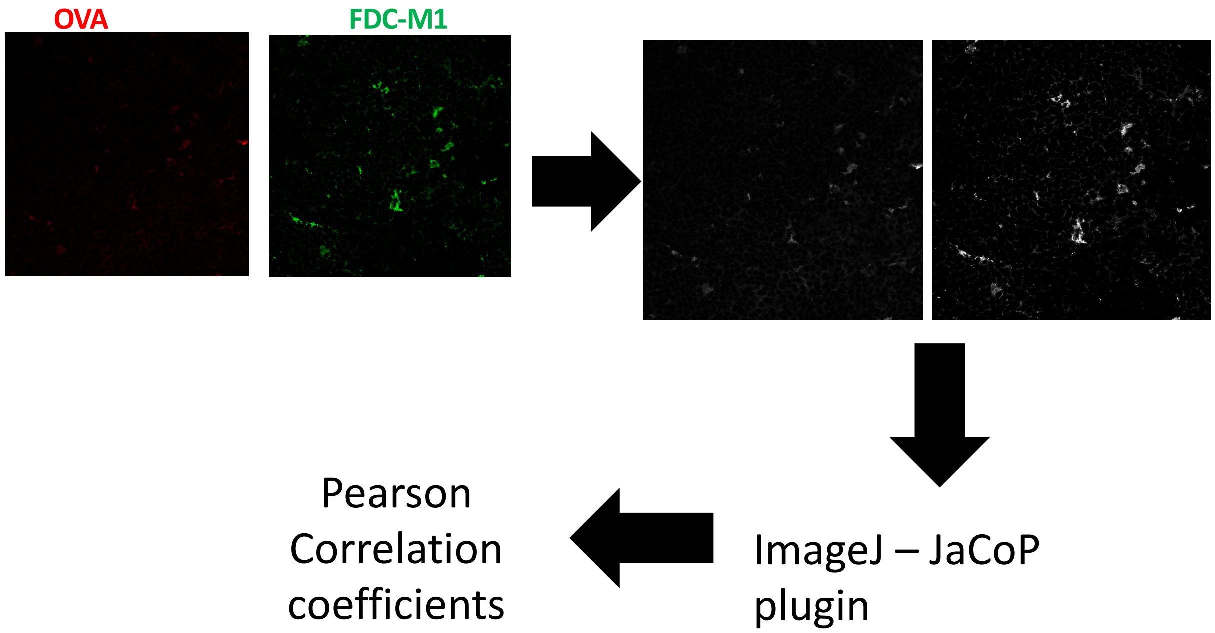

Supplemental Figure 5: Pearson correlation coefficient analysis. Images were imported into ImageJ and then changed to a 16-bit image. The plugin JaCoP was then utilized to determine Pearson correlation coefficients for OVA and FDCs.

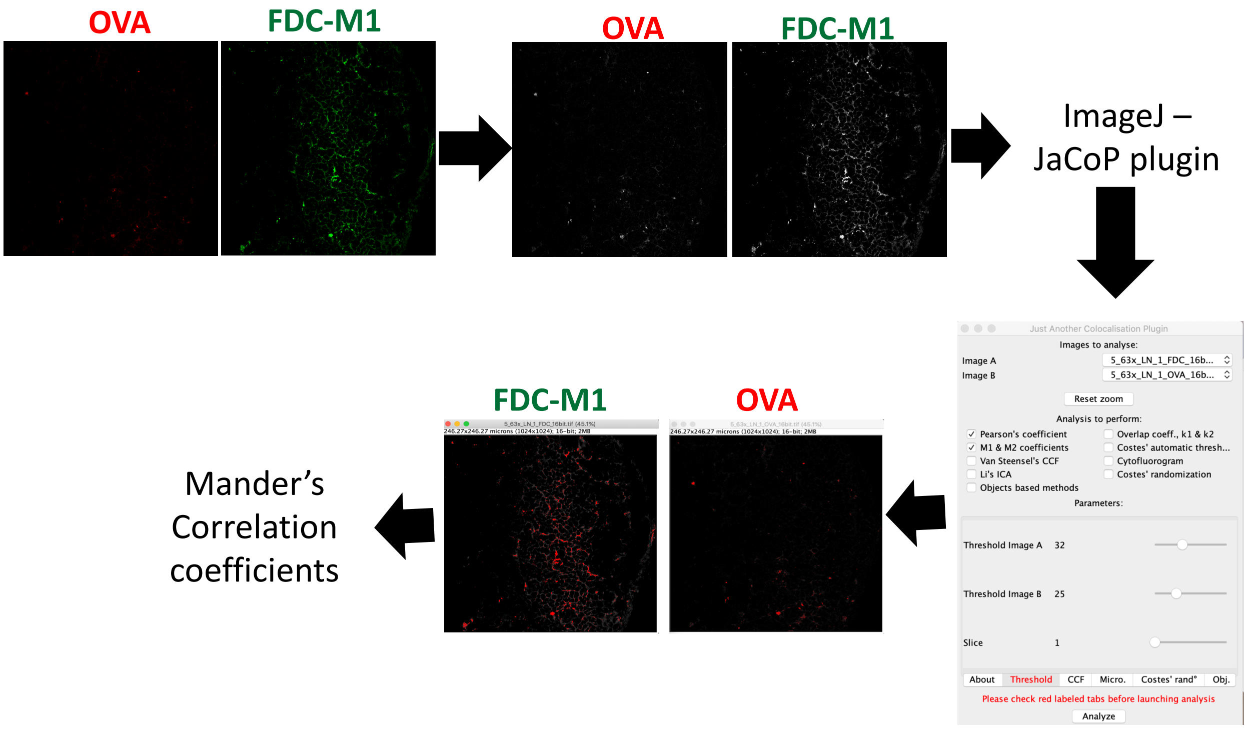

Supplemental Figure 6: Pearson correlation coefficient analysis. Images were imported into ImageJ and then changed to a 16-bit image. The plugin JaCoP was then utilized to determine Manders correlation coefficient to determine what percent of OVA was associated with either DCs or FDCs.
